# Oral Hygiene Instructions and Methods: A Comparative Survey of European General Dentists, Periodontists and Dental Hygienists

**DOI:** 10.3290/j.ohpd.b1749661

**Published:** 2021-07-15

**Authors:** Valentin Garyga, Laurence Seidel, Gilles Gagnot, Michèle Reners, France Lambert

**Affiliations:** a Dentist in Private Practice, Lyon, France; Assistant Professor, Faculty of Odontology, University of Lyon, Lyon, France; Dental Teaching Hospital, Lyon University Hospital, Lyon, France; Department of Periodontology and Oral Surgery, Faculty of Medicine, University of Liège, Belgium. Wrote the manuscript.; b Biostatistician, Department of Biostatistics and Medico-economic Information, University Hospital of Liège, Belgium. Performed statistical evaluation.; c Dentist in Private practice, Vitré, France. Idea, hypothesis, and survey realisation.; d Dentist in Private Practice, Liège, Belgium; Lecturer, University of Liège, Department of Periodontology and Oral Surgery, Faculty of Medicine, University of Liege, Belgium. Idea, hypothesis, and survey realisation.; e Professor and Head, Department of Periodontology, Oral and Implant Surgery, Faculty of Medicine, University of Liège, Belgium; Dental Biomaterials Research Unit (d-BRU), Faculty of Medicine, University of Liège, Belgium. Idea, hypothesis, survey realisation, wrote the manuscript.

**Keywords:** dental hygienist, oral hygiene, patient education as topic, periodontal diseases, periodontists, surveys and questionnaires

## Abstract

**Purpose:**

To study the practices of general dentists, periodontists and dental hygienists who are members of the European Federation of Periodontology, regarding oral hygiene education, plaque control assessment, recommended dental and interdental hygiene tools, and antimicrobial agents.

**Materials and Methods:**

A web-based survey was sent to 13,622 members of the European Federation of Periodontology (EFP) through its 29 national member societies. It targeted general dentists (GD), specialists in periodontology (DSP) and dental hygienists (DH). Data were collected between 24 April and 17 May 2015. A data-driven statistical analysis was conducted and differences between professions were explored.

**Results:**

A total of 2076 answers were collected. Only the 2009 answers originating from GD, DSP and DH were analysed (67 answers originated from other professions and were excluded). Among those 2009 respondents, 43.2% were DSP and 37.2% were GD. Overall, DH, DSP and GD reported spending 17.1 minutes for the initial teaching of OH, with differences between professions (p < 0.0001). DH, GD and DSP exhibited differences in the type of toothbrushes they recommend (p < 0.0001). DSP recommended electric and manual toothbrushes (TB) equally. DH predominantly recommended electric TB (56.8%). Overall, 95% of DH, DSP and GD recommended interdental brushes, with no statistically significant differences between professions. Low concentration chlorhexidine was considered the most relevant antimicrobial agent for daily oral care of periodontitis patients. Half of GD prescribed antimicrobial mouthrinses for long-term use in 70%–100% of their patients with periodontitis.

**Conclusion:**

EFP-affiliated practitioners allocate a significant amount of time to educating patients on oral health. Their practices are mostly in line with the current scientific evidence. Some discrepancies were found between the different professions. Similar surveys could be conducted over time to monitor the evolution of practices.

Oral diseases are among the most common conditions of humankind: 3.5 billion persons worldwide suffered from untreated oral conditions in 2015.^24^ Notably, 50% of the adult population worldwide suffers from some form of periodontal disease.^12^ Dental caries, periodontitis and oral cancer are a major burden for public health^40^ that may be avoided with primary or secondary prevention.^4^

The accumulation of dental plaque has been proven as a main risk factor for the development and progression of periodontal diseases.^9,31^ Dental plaque is a bacterial biofilm with an estimated 1200 predominant bacterial species whose exact composition varies between patients and tooth sites.^25,53^ Regular and effective supragingival plaque control, notably via interdental care, can alter the composition of the periodontal pocket microbiota, lowering the percentage of periodontopathogenic bacteria.^11,21,46^ However, a significant portion of the population suffers from persistent gingivitis, which is a risk factor for periodontal attachment loss and tooth loss.^29^ In patients treated for periodontal diseases, failure to maintain a good oral health (OH) can lead to treatment failure in the long term.^13^ Hence, patient motivation and instruction is critical for OH.

Strategies to improve patient knowledge and compliance are intensively explored in contemporary research.^2^ However, the range of patient education interventions is large,^1^ and the lack of scientific consensus fosters heterogeneity. Also, the composition of the dental team varies across European countries. Twenty-three out of 32 European countries recognise the profession of dental hygienist.^27^ The roles, competences and employment status of members of the dental team may have a profound impact on how dental care is organised, and many models can be envisioned.^10,20,47^ A lack of practice-based data makes it difficult to compare attitudes among European professionals.

The purpose of this study was to conduct a survey across members of the European Federation of Periodontology (EFP) to investigate modalities of oral hygiene education, plaque control assessment, recommended dental and interdental hygiene tools, and the use of antimicrobial agents. The results were compared between general dentists (GD), specialists in periodontology (DSP) and dental hygienists (DH). The data provided by respondents may help bridge the gap between practitioners’ practices and evidence supported by the literature.

## Materials and Methods

### Study Population and Methodology, Study Design and Questionnaire

A cross-sectional European web-based survey was held among dental care providers across the 29 national periodontal societies (NPS) of the EFP. The surveyed population comprised GD, DSP, DH and assistants in dental hygiene, who were members of all 29 EFP NPS. The ethics committee of Liège University hospital in Liège, Belgium was consulted and it ruled that approval was not needed.

The online survey was coordinated by an independent market research institute (Dedicated; Ixelles, Belgium). By sending an invitation e-mail, each NPS communicated to their affiliates the online hyperlink for completing the survey. This approach guaranteed the confidentiality of the affiliates’ contact data.

A total of 13,622 practitioners were contacted via a personalised e-mail, inviting them to take part in the survey and to complete the online questionnaire. A reminder e-mail was sent 7 days later if the person had not responded to the first invitation. The reported data were anonymous and confidential. The questionnaire could only be filled out once by each practitioner. This method is considered valid for data collection.^42^ Data were collected between 24 April and 17 May 2015.

The 55-question survey (average duration: 10 minutes) explored OH and modalities of treatment for periodontal and peri-implant diseases. The present report focuses on OH, based on a set of 22 questions.

Eleven questions explored the profile and demographics of respondents (sample description). Another set of 11 questions explored oral hygiene education, plaque control assessment, recommended dental and interdental hygiene tools, and the use of antimicrobial agents. Most questions were in a multiple-choice format. For some, multiple answers could be selected. For analysis, frequencies of certain treatments were assessed by means of Likert scales (i.e. rating scales). These 22 questions are summarised in Table 1.

**Table 1:** The set of 22 questions considered for this report

		Type of questions
**Sample description**		
	Country	MCQ + Open
	Profession	MCQ
	Type of clinic (e.g. private)	MCQ
	Size of clinic (e.g. group practice)	MCQ
	Place of practice (e.g. urban)	MCQ
	Date of birth	Date
	Years of experience	Number
	Number of patients per day	MCQ
	Days of work per week	MCQ
	Involvement in teaching	MCQ
Specific questions		
Oral hygiene instruction	Time allocated	Number
	Professionals involved	MCQ
	Explanatory leaflet provided	Yes / No
Plaque control assessment	Frequency	Likert scale
	Tools used	MCQ + Open
Dental and interdental hygiene	Kind of toothbrush	MCQ
	Tools for interdental care	MCQ + Open
Antimicrobials	Toothpaste with AM – gingivitis	Likert scale
	Toothpaste with AM – periodontitis	Likert scale
	Mouthrinse with AM – periodontitis	Likert scale
	AM considered relevant – periodontitis	MCQ

The remaining questions, which address treatment modalities of periodontitis and peri-implantitis, will be made available in a separate report. The questionnaire was translated from English into 4 languages (French, German, Italian, Spanish).

### Statistical Analysis

**Fig 1:**
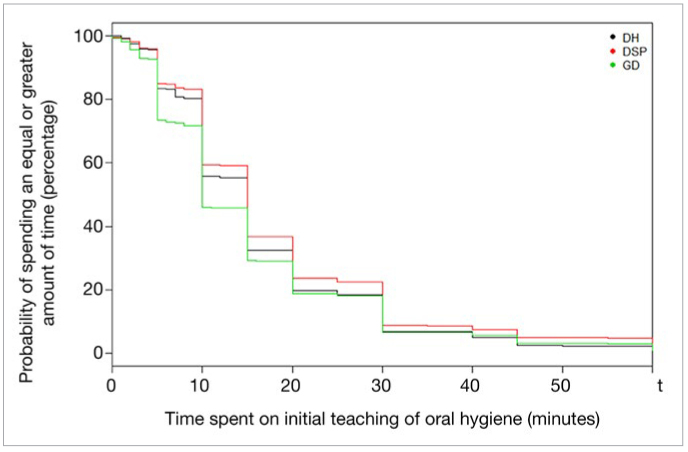
Kaplan-Meier plots of time spent for initial oral hygiene education.

**Fig 2:**
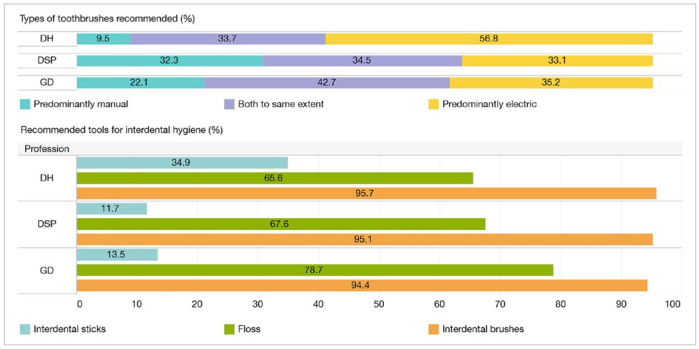
Dental and interdental hygiene tools recommended by survey participants.

**Fig 3:**
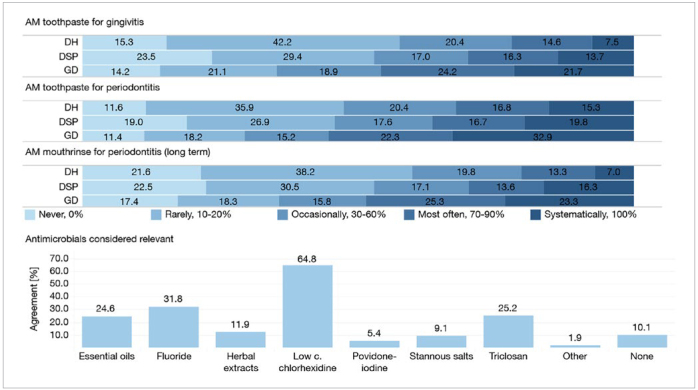
Antimicrobial prescription patterns.

The database was checked for consistency and cleared of patently visible errors. Descriptive statistics were computed for all variables. The chi-squared test as well as multinomial and ordinal logistic regressions were used to explore the impact of profession on respondents’ answers. The Kaplan-Meier curve and the log-rank test were used to represent and compare duration of initial teaching between professions. When comparing the differences between professions, only answers from GD, DSP and DH were considered for the analysis.

p-values were considered statistically significant at the 5% level (p < 0.05). SAS version 9.4 statistical software (SAS Institute; Cary, NC, USA) was used for descriptive statistics, chi-squared tests, multinomial and logistic regression, and R version 3.2.2 software (Vienna, Austria) was used for the log-rank testand to generate the Kaplan-Meier curve (Fig 1). Tableau data visualization software, version 2018.2 (Tableau Software; Seattle, WA, USA), was used to create Figs 2 and 3.

## Results

### Demographics

Out of 13,622 sent questionnaires, a total of 2079 answers were collected. Therefore, the raw response rate was of 15.2%. In addition to the 2009 GD, DSP and DH who completed the survey, 67 other healthcare providers (e.g. other specialist dentists, dental assistants, dental students) also answered the survey. Seven countries accounted for 1203 (57.9%) of answers (France, Italy, the Netherlands, Spain, Sweden, Belgium and the United Kingdom). For the rest of this report, only answers by GD, DSP and DH are considered and reported; thus, all calculations were performed using a sample size of 2009.

Gender distribution among GD, DSP and DH was approximately 1:1 (female: 53.1% [n = 1066], male: 46.9% [n = 943]). It is of note that DH were mainly females (92.5%, n = 368). The mean age of GD, DSP and DH was 44 years (SD=12). Respondents had on average 18 years of experience (SD=11). Looking at the profession of the respondents, 398 (19.8%) were DH, 863 (43%) were DSP, and 748 (37.2%) were GD. 1636 (81.4%) were private practitioners. Also, 459 (22.8%) of respondents worked in a university clinic. The proportion of DH working in a university clinic was lower than that of GD (OR = 0.16, p < 0.0001), while the proportion of DSP working in a university clinic was higher than that of GD (OR = 4.4, p < 0.0001). 838 respondents (41.7%) were involved in the teaching of periodontology; among them, 472 (56.3%) were teaching in a university setting. Finally, 1650 (82.1%) participants reported working in an urban area, and DSP worked more often in urban areas than did GD (OR = 2.9, p < 0.0001).

Demographic characteristics of the GD, DSP and DH sample are reported in Table 2.

**Table 2:** Demographics of the surveyed population

	Category	Mean ± SD	Minimum	Q1	Median	Q3	Maximum
Age (years)	DSP	43 ± 10.8	23.5	34.3	41.5	51.3	72.1
	GD	46.6 ± 12	22.7	36.3	47.9	56.8	85.3
	DH	42.1 ± 11.7	22.3	31.2	42.2	52	70.6
Experience (years)	DSP	17.6 ± 10.7	1	8	16	26	49
	GD	21.1 ± 12	1	10	22	31	60
	DH	15.6 ± 11.3	1	5	13.5	25	41
	Category			Frequency (%)	Odds ratio		
					DSP v. GD	DH v. GD	
Profession	DSP			863 (41.6)			
	GD			748 (36)			
	DH			398 (19.2)			
	Other			67 (3.2)			
Gender	DSP, GD and DH		Male	943 (46.9)			
			Female	1066 (53.1)			
		DSP	Male	469 (54.3)			
			Female	394 (47.7)			
		GD	Male	444 (59.4)			
			Female	304 (40.6)			
		DH	Male	30 (7.5)			
			Female	368 (92.5)			
Type of clinic	Private			1604 (79.8)	0.42***	0.067***	
	Public			288 (14.3)	1.13 (NS)	1.32 (NS)	
	University			421 (21.0)	4.41***	0.17***	
	Other			43 (2.1)	1.05 (NS)	0.60 (NS)	
Place of practice	Urban			1650 (82.1)	2.92***	0.76 (NS)	
	Semi-rural			333 (16.6)	0.96 (NS)	0.96 (NS)	
	Rural			111 (5.5)	0.78 (NS)	0.57 (NS)	
Country of origin	7 countries			1175 (58.5)			
	France			319 (15.9)			
	Italy			192 (9.6)			
	Netherlands			181 (9.0)			
	Spain			165 (8.2)			
	Belgium			112 (5.6)			
	Sweden			110 (5.5)			
	Finland			96 (4.8)			
	All other countries			834 (41.5)			

*p < 0.05, **p < 0.001, ***p < 0.0001. Odds ratio were computed by multinomial logistic regression. GD: general dentist, DSP: dental specialist in periodontology; DH: dental hygienist. NS: non-significant. Repondents whose profession was listed as ‘other’ were not considered for analysis. Three outliers (1 GD and 2 DSP) were excluded from calculations relating to age as they clearly reflected input errors.

### Work Organization

1322 (65.8%) professionals saw fewer than 15 patients a day. There was a statistically significant difference in the number of patients per day between professions (p < 0.0001). GD and DSP saw more patients than did DH (OR = 5.8 and OR = 2.9, respectively, both p < 0.0001) and DSP saw fewer patients than did GD (OR = 0.51, p < 0.0001).

1664 respondents (82.8%) worked at least 4 days a week. There were also statistically significant differences among professions (p < 0.0001). DSP and GD worked more than DH (OR = 2.3 and OR = 1.9, respectively, both p < 0.0001), but no difference was found between DSP and GD.

Full results are reported in Table 3.

**Table 3:** Work organisation

Profession	Number of patients per day (χ^2^***)			Days worked per week (χ^2^***)		
	<15 (%)	15-25 (%)	>25 (%)	<2 (%)	2-4 (%)	≥4 (%)
DH	343 (86.2)	51 (12.8)	4 (1)	16 (4)	89 (22.3)	293 (73.6)
DSP	594 (68.8)	217 (25.1)	52 (5)	14 (1.6)	103 (11.9)	746 (86.4)
GD	385 (51.5)	309 (41.3)	54 (7.2)	11 (1.5)	112 (15)	625 (83.6)
	More patients per day			More days worked per week		
	Odds ratio‡	Wald 95% CI		Odds ratio‡	Wald 95% CI	
DSP > GD	0.51***	0.41–0.62		1.25 (NS)	0.95–1.64	
DSP > DH	2.91***	2.12–4.00		2.30***	1.71–3.09	
GD > DH	5.78***	4.19–7.91		1.85***	1.38–2.48	

*p < 0.05, **p < 0.001, ***p < 0.0001. ‡ Ordinal logistic regression. χ^2^: chi-squared test (overall). NS: non-significant.

### Oral Hygiene Education (OHE)

Respondents reported spending a median of 15 min (mean: 17.1, IQR: 10-20) for the initial teaching of OH. However, there were significant differences between respondents (SD=14.3; minimum: 0 for 11 respondents; maximum: 120 for 5 respondents) and between professions (p < 0.0001). DSP spent significantly more time teaching than did GD (DH: 15 min, DSP: 15 min, GD: 10 min, p < 0.05), but no difference was found between DSP and DH, nor between GD and DH. Kaplan-Meier curves of time spent for OHE are displayed and interpreted in Fig 1. More data for each profession in available in Annex 1.

All professions believed they were the most involved in a patient’s initial OH instruction: 98.2% (n = 391) of DH thought they were the most involved in OHE. The rates are 61.4% (n = 530) for DSP and 61.1% (n = 457) for GD. 1834 respondents (91.3%) provide some of their patients with an information leaflet on periodontal disease and its treatment. 51.6% (n = 445) of DSP, 35.2% (n = 140) of DH and 33.4% (n = 250) of GD provide leaflets for all their patients. Only 175 respondents (8.7%) never provide such a document.

### Plaque Control Assessment

701 (81.2%) DSP declared performing a plaque control assessment (PCA) at each non-surgical appointment. The regularity of PCA differed among DH, DSP and GD (p < 0.0001). The proportion of DSP performing regular PCA was higher than for GD and DH (OR = 2.0 and OR = 1.8, respectively, both p < 0.0001). No statistically significant difference was found between GD and DH.

Respondents were asked to provide further information about the tools they use for PCA. 1017 (50.6%) declared using plaque disclosing solution. 193 (9.6%) declared using an intra-oral camera.

PCA results are summarised in Table 4.

**Table 4:** Frequency of oral hygiene diagnosis

Profession	Frequency of plaque control assessments (PCA) (χ^2^***)		
	Initial treatment only (%)	Sometimes (%)	Each non surgical appointment (%)
DH	49 (12.3)	66 (16.6)	283 (71.1)
DSP	68 (7.9)	94 (10.9)	701 (81.2)
GD	84 (11.2)	157 (21.0)	507 (67.8)
	More frequent PCA		
	Odds ratio‡	Wald 95% CI	
DSP > GD	1.98***	1.57–2.49	
DSP > DH	1.76***	1.34–2.31	
GD > DH	0.89 (NS)	0.68–1.15	

*p < 0.05, **p < 0.001, ***p < 0.0001. ‡ Ordinal logistic regression. χ^2^: chi-squared test (overall). NS: non-significant.

### Dental and Interdental Hygiene Tools

Respondents were asked about the kind of toothbrushes (TB) (electric or manual) and interdental hygiene tools they usually recommend to patients. DH, DSP and GD had different attitudes regarding the kind of TB they recommend to their patients (p < 0.0001). DH predominantly recommended electric TB (56.8%, n = 226), while DSP recommended electric and manual devices equally. Overall, DH recommended electric TB more than GD did (OR = 1.8, p < 0.0001). DSP recommended electric TB less than GD did (OR = 0.55, p < 0.0001).

For interproximal cleaning, 95% of respondents (n = 1908) recommended interdental brushes with no differences between professions. Also, 78.7% (n = 569) of GD recommended dental floss and 34.9% (n = 139) of DH recommended interdental sticks (wood or plastic). Dental floss is less often recommended by both DH and DSP compared to GD (OR = 0.54 and OR = 0.56, respectively, p < 0.0001), and DH prescribe interdental sticks more often than GD do (OR = 3.2, p < 0.0001). Data regarding the dental and interdental hygiene tools are depicted in Fig 2.

### Antimicrobial (AM) Agents

Low-concentration chlorhexidine (LC-CHX) was considered the most (64.8%, n = 1302) relevant AM agent for daily oral care of periodontitis patients. Fluoride (FL), triclosan (TCS) and essential oils (EO) were considered relevant by 31.8% (n = 639), 25% (n = 507) and 24.6% (n = 495) of respondents, respectively. Also, 10.1% (n = 202) did not consider any AM agent relevant.

Regarding AM mouthrinses (MR), GD were the most likely to prescribe the long-term use of an AM MR for periodontitis, while DH were the least likely to do so (OR = 2.4 when GD are compared to DH, p < 0.0001). 363 (48.5%) of GD prescribe AM MR for long-term use in periodontitis for 70%-100% of their patients.

Regarding the use of AM toothpastes (TP), DSP and DH do not often prescribe them. No difference was found between DH and DSP in terms of their AM TP prescription patterns. On the other hand, GD prescribed AM TP both for gingivitis and periodontitis the most often (compared to DH: OR = 2.2, p < 0.0001 for gingivitis and OR = 2.1, p < 0.0001 for periodontitis). Indeed, 45.9% (n = 343) and 55.2% (n = 413) of GD routinely prescribe AM TP (over 70% of the time) to treat gingivitis or periodontitis, respectively. Data related to TP, MR and AM agents are summarised in Fig 3.

## Discussion

To the authors’ best knowledge, this work is the first wide-scale survey targeted at European dental care providers gathering practice-based data on periodontology. The questions addressed key elements of the periodontal clinical work: prophylaxis interventions, OHE and the prescription of AM agents in periodontology.

### Methodology and Representativity

This work featured a very large sample (over 13,622 EFP members were contacted) and the use of a proven data collection method (web-based platform and one e-mail reminder).^16,18^ The response rate of 15.2% was in line with another 100% web-based survey of US periodontists reported in the literature.^38^ Phone reminders or mail invitations^39^ may have yielded higher response rates, but they were not possible given the wide scale of the survey. The low response rate, however, does not necessarily induce an absence of reliability. Respondents had similar working experience (mean: 18 years, IQR: 8-28 years) and age (mean: 44 years, IQR: 34-54 years) among those dentists practicing in France,^35^ the Netherlands,^23^ Spain,^30^ Sweden,^36^ and Belgium.^8^ 44.2% (n = 887) of all respondents originated from these five countries. This suggests that the demographics of respondents can be considered fairly representative of the population of interest in these five countries.

Potential limits of this study include its external validity and the difficulty of appraising a complex clinical practice with a 10-minute questionnaire. Respondents of seven countries (France, Italy, The Netherlands, Spain, Sweden, Belgium, The United Kingdom) accounted for 57.9% (n = 1203) of answers. However, dentists from these countries account for a little over half of all practicing or registered dentists within the European Union (EU) and European Economic Area (EEA) (estimate: 350,000).^15^ This mismatch may be related to the nationalities of the investigators (French and Belgian) and/or to the activity levels of some EFP NPS.

Another limitation is the distinction between GD and DSP. For instance, 69 out of 319 French respondents defined themselves as DSP, although the specialty of periodontology is not formally recognised in France. This data is self-reported and there was no possibility for the survey authors to verify respondents’ qualifications. Such a limitation is frequent in survey research. This is also produced by the legislative environment, because the specialty of periodontology is not listed in the European Union Professional Qualifications Directive; thus, every Member State of the EU can decide to formally recognise it or not. While the specialty of periodontology is not universally recognised in Europe, it does not preclude individual practitioners from gaining an equivalent qualification and/or limit their scope of practice to periodontology. As such, irrespective of the country, some of these ‘ad-hoc specialists’ might be on par with regular board- or state-certified specialists. Furthermore, requaliying GDP as DSP would also induce bias in the statistical analysis. It was therefore decided to analyse the data as is.

Also, 23.5% (n = 472) of respondents reported teaching at a university, while recent European figures state that only 2.6% of dentists work in universities, with no available information regarding full-time or part-time positions.^27^ On the other hand, 81.4% (n = 1636) of respondents work in private clinics: the results are fairly consistent with the estimated 89% of European dentists working in private clinics (including liberal practice).^27^ Given such limitations, generalising our results may only be appropriate considering a target population of affiliates of the EFP who are more likely to be knowledgeable about periodontal science. Caution is advised when generalising these findings to all European dental care providers.

### Oral Hygiene Instructions

With an average of 17.1 minutes spent on OHE, the population of the present survey allocate a significant amount of their time to educating their patients. Only an anecdotal number of respondents did not spend any time on OH education (OHE): 6 GD and 5 DSP. Overall, DSP allocated more time than GD did. No difference was found between DSP and DH. Also, it appeared that DSP assessed the patient’s plaque control more often than did GD and DH. GD were more likely than DSP and DH to see more patients per day, and hence probably had shorter appointments. To the authors’ best knowledge, there are no guidelines on the duration for OHE, but the collective evidence establishes that patient instruction and motivation, regular check-up visits and professional feedback and reinforcement are critical for successful prevention and treatment of periodontal diseases. By spending more time on OHE, DSP comply with such evidence. Frequent recalls increase patient compliance in the context of OH motivation,^5,17^ and close monitoring, notably by plaque control assessment, is essential for the prevention of relapse. It has been reported that patients forget 40%-80% of information given during a medical consultation^26,52^ and up to 50% of what they remember is incorrect.^3^ By providing an information leaflet about periodontal diseases and their treatment, 9 out of 10 respondents gave their patients a better chance at reminding them of their advice and complying correctly with their new OH routine.

### Tools for Oral Hygiene

A Cochrane systematic review found that while powered toothbrushes perform slightly better than manual toothbrushes for plaque removal, the clinical implications of this remain unclear.^54^ The current paradigm is that OH instruction should be tailored to each individual patient on the basis of her/his personal needs and other factors,^28^ there is no reason to introduce a specific toothbrushing technique with each new patient.^34^

Regarding proximal care, with 95% (n = 1908) of professionals recommending interdental brushes, the situation appears very satisfactory. EFP affiliates are in clear alignment with the state of the evidence: interdental brushes are the most effective tool for interproximal plaque removal.^11,22,46^ However, interdental sticks remain largely recommended by hygienists (34.9%, n = 139), even though they are proven to be less effective than interdental brushes for plaque removal.^44,46^ As such, the present results suggest the target population of EFP-affiliated care providers have a strong culture of oral health education with sound scientific grounds although some discrepancies persist between GD, DSP and DH.

### Antimicrobials

Several antimicrobial agents are available in mouthrinses (MR) and toothpastes (TP) and have shown their added value for adjunct chemical plaque control.^45^ Recommendations from the EFP workshop on prevention state that anti-plaque chemical agents delivered in a MR or TP format, adjunctive to toothbrushing, are beneficial.^9^ Decisions should consider the economic cost, adverse events associated with long-term use of AM agents and country-specific regulations or guidelines.^9^

48.5% (n = 363) of GD declared prescribing AM MR 70%-100% of the time for long-term use in periodontitis patients. LC-CHX was considered the most relevant AM agent by respondents (64.8%, n = 1302). CHX allows better reduction of plaque (-33%) and gingivitis (-26%) compared to placebo.^50^ A systematic review concluded that both low- and high-concentration CHX mouthrinses provided significant reductions in plaque and gingivitis.^45^ 0.12% or 0.2% showed the best results and there is evidence for their safety in long-term use. Still, because of staining, other products may be preferred for long-term use, such as LC-CHX. The association of agents, such as 0.05% cetylpyridinium chloride (CPC) and 0.05% CHX somehow compensates for the lower concentration of CHX. Staining can still occur, but at lower levels.

EO were selected by 24.6% (n = 495) of respondents. EO are mostly delivered in MR and have a proven efficacy for plaque reduction and gingivitis reduction,^19^ including non-alcoholic formulae.^48^ EO-based MR appear to be an alternative to CHX for long-term maintenance use.^49^

45.9% (n = 343) and 55.2% (n = 413) of GD prescribed AM TP 70%-100% of the time for gingivitis and periodontitis, respectively.

FL was selected by 31.8% (n = 639) of respondents. Periodontal patients are particularly at risk of root caries,^6^ and TP containing 5000 ppm F^-^ are effective for their non-invasive treatment.^51^

TCS (triclosan) was selected by 25% (n = 507) of respondents. Its value for adjunct plaque control is strongly supported by three systematic reviews in the literature.^45^ A Cochrane review estimates its effect of 22% reduction in plaque, 22% reduction in gingival inflammation, and 48% reduction in bleeding scores.^41^

Stannous fluoride (SnF), chosen by 9.1% (n = 182) of respondents, exhibited no clinically relevant difference in plaque scores or gingival inflammation when compared to TCS in TP.^43^ In the past, staining with SnF was a concern, but it is now used in combination with sodium hexametaphosphate (SHMP),^7,32^ and for that particular formulation, staining seems reduced.^33^

TCS-based TP are also an alternative, as long-term data demonstrate that TCS-copolymer and SnF-SHMP show equivalent results in periodontitis risk progression.^37^ More evidence is available for TCS-copolymer, with 19 randomised controlled trials of at least 6 months of follow-up and only 3 such studies for SnF-SHMP.^14^

GD tended to prescribe more AM TP than did DSP and DH, regardless of the diagnosis (gingivitis/periodontitis). DSP and DH may place more importance on the mechanical aspects of plaque control, as they spend more time on OHE and can thus better instruct the patient. Overall, the results of this study are in line with a recent network meta-analysis which found that EO-based and CHX-based formulae for MR, as well as the TCS-containing formulae for TP, had the greatest effects on supragingival plaque control.^14^ TP and MR were analysed separately, and products with the larger number of available studies and with evaluation of the particular indices included in the analysis yielded better results.

### Further Investigations

The present results allow a good representation of the involvement of European periodontal practitioners in patient education and prevention. Exploring their attitudes towards non-surgical and surgical management of periodontal and peri-implant diseases would lead to an even more complete depiction of the state of periodontal practice in Europe. Additionally, as EU Member States are in charge of the main regulatory aspects for health professionals, there is a potential for heterogeneity in education, training and practice organisation. Therefore, further investigations should be conducted at the national level to provide in-depth analysis of oral health systems.

## Conclusion

This survey generated a substantial body of data concerning the views and practice of European periodontists, dentists and hygienists in key domains of patient education and periodontal prevention. The present work demonstrated the feasibility of international, large scale surveys of European periodontal practitioners.

EFP-affiliated practitioners seem fill their roles as patient educators very conscientiously. They allocate a significant amount of time to patient education and offer advice on daily dental and interdental care in line with the state of scientific evidence. Some discrepancies were found between the different professions, and hypotheses were proposed. Further investigations with wide-scale surveys could explore other populations of dental care providers.

## Appendix

**Annex 1:** Time spent for initial oral hygiene education

	Time spent for initial oral hygiene education							
	N	Mean	Std	Min	Q1	Median	Q3	Max
Profession	398	16.53	11.76	1.00	10.00	15.00	20.00	60.00
Dental hygienist								
Dentist specialist in periodontology	863	18.64	15.57	0.00	10.00	15.00	20.00	120.00
General dentist	748	15.68	13.97	0.00	5.00	10.00	20.00	120.00
